# Factors associated with non-use of condoms among heterosexually-active single people in Germany: Results from the first representative, population-based German health and sexuality survey (GeSiD)

**DOI:** 10.1177/09564624221100309

**Published:** 2022-05-25

**Authors:** Susanne Cerwenka, Christian Wiessner, Catherine H Mercer, Silja Matthiesen, Laura Pietras, Ursula von Rüden, Arne Dekker, Peer Briken

**Affiliations:** 1 Center for Psychosocial Medicine, Sexual Medicine and Forensic Psychiatry, Institute for Sex Research, 37734 University Medical Center Hamburg-Eppendorf, Hamburg, Germany; 2 Centre for Population Research in Sexual Health and HIV, Institute for Global Health, University College London, London, UK; 3 Bundeszentrale für gesundheitliche Aufklärung (BZgA), Köln, Germany

**Keywords:** Condoms, heterosexuality, safer sex, sexual partners, German health and sexuality survey-study

## Abstract

**Background:** Against the backdrop of rising STI incidence among the heterosexual population, sexually active single people are at particularly high STI transmission risk. Gaining insight into circumstances related to condoms non-use in this population is therefore important for developing effective health interventions. **Methods:** The nationally-representative survey, GeSiD (German Health and Sexuality Survey) undertaken 2018–2019, interviewed 4,955 people aged 18–75 years. A total of 343 heterosexually-active single participants answered a question about condom use at last sex. Data on sociodemographic characteristics, sexual behaviours and circumstances of last sex were analysed to identify independently associated factors. **Results:** Condom non-use at last sex was reported more commonly by participants aged >35 years than by younger participants (48.5 vs 33.7%, respectively) and more likely among longer relationships (adjusted odds ratio [AOR]: 2.43) or early loving relationships (AOR: 3.59) than in one-night-stands. It was also associated with not discussing using condoms before sex (AOR: 6.50) and with reporting non-use of condoms at sexual debut (AOR: 4.75). **Conclusions:** Non-use of condoms is a common STI risk behaviour among heterosexually-active single people in Germany and so needs promoting from sexual debut throughout the life course, regardless of relationship type and age, but particularly among middle-aged and older people.

## Introduction

Like other European countries, such as the Netherlands,^1^ there has been an increase in the number of HIV diagnoses through heterosexual transmission in recent years, as well as considerable increases in the numbers of diagnoses of other STIs, such as chlamydia and HPV, especially in young women as part of routine reproductive healthcare check-ups.^2,3^ Non-use of condoms is a key risk factor in STI/HIV transmission particularly in the context of partner change,^4–6^ and with new partners whose sexual history and STI testing practice is less likely to be known.

To date, research regarding factors associated with the non-use of condoms has mainly focused on men who have sex with men (MSM) as a population who are at particularly high risk of STI/HIV transmission, while the heterosexual population has gained increased attention in empirical research only in recent years. This research shows that single heterosexuals who are casually dating are more likely to use condoms than those in a committed relationship or with a steady sex-partner.^4,6–11^ While some other countries have population-based survey data to examine the context of non-use of condoms (e.g. 4,5,8,11,12), this has not been the case for Germany. The present study, to our knowledge, is the first to investigate factors associated with the non-use of condoms in the German population, focusing on heterosexually-active single people, in terms of the key sociodemographic characteristics, sexual behaviours and aspects related to the relational/situational features of the sexual encounter.

## Materials and methods

### Study and data collection

The “German Health and Sexuality Survey - GeSiD” is a cross-sectional study with a population representative sampling scheme conducted by the Institute for Sex Research, Sexual Medicine and Forensic Psychiatry at the University Medical Centre Hamburg-Eppendorf in collaboration with the social research institute KANTAR and funded by the Federal Centre for Health Education (BZgA). The study was approved by the ethics committee of the Hamburg Psychotherapy Association (“*Hamburger Psychotherapeutenkammer”*).

Based on a two-stage random sample design with names and addresses drawn from 200 “sample points” (usually coinciding with the residents’ registration offices) throughout the entire Federal Republic of Germany, in the time period 2018 to 2019. Altogether, 4,955 individuals (2,336 men and 2,619 women) aged between 18 and 75 years participated, which included oversampling those aged 18–35, accounting for 37.7% (*n* = 1,869) of the sample. The response rate, according to AAPOR criteria,^13^ was 30.2% (AAPOR Response Rate 4).

The survey was conducted by means of computer-assisted personal interviews (CAPI) in the participant’s own home, and included an extensive self-completion section (computer-assisted self-interview, CASI). Before the start of the interview, respondents confirmed that they had been informed about the goals of the study, anonymisation and data protection, and the voluntary nature of participation by providing a written declaration of consent.

The questionnaire, which had previously been tested in a pilot study,^14^ included over 260 questions on a range of topics including various sexuality-related topics, developed following extensive research of similar studies internationally, as well as the WHO Indicators of Sexual Health.^15^ Women were interviewed by women and men by men. The mean duration of the interviews was 51 min (median = 48, range 19–208 min).

### Population of interest

The present analyses focus on the subsample of heterosexually-active single people, defined as those who reported no current relationship, that they had been single for at least 1 year prior to interview, and who stated at least one sexual encounter with someone of the opposite sex during this time.

### Primary outcome

#### Non-use of condoms at last sex

Participants were asked whether they had used any contraceptives at last sex and if so, which. The question had been adapted from earlier German studies^16^ and offered multiple answer options. One of these options was condom use. Answers were dichotomised with regard to the non-use of condoms at last sex into “condoms used” and “non-use of condoms” (no contraception or other type of contraception).

### Factors hypothesised to be associated with non-use of condoms

#### Sociodemographic characteristics

Categorised variables were included on the basis of the information provided by the respondents on: their age at the time of the interview (18–35 years; 36–75 years), level of education (“low”; “medium”; “high”; broadly corresponding to: no school-leaving qualification (yet) or lower secondary school; secondary school; university entrance qualification or academic degree) and migration background (“no”; “yes” [participant and/or parent born in another country]).

#### Sexual behaviour and sexual history

##### Type of sexual practices during the latest sex

Participants were asked in the CASI about the sexual practices they had performed at last sex (vaginal intercourse; oral sex; anal sex; other genital contact, e.g. manual contact). Multiple response options were categorized into “vaginal intercourse only”, “vaginal intercourse and oral sex” and “vaginal intercourse combined with oral sex and other genital contacts”. Other types and combinations of sexual practices were pooled into one category due to small numbers (*n* < 30).

##### Number of opposite-sex partners during the past year

Women were asked how many men they had had sex with, men were asked how many women they had had sex with during the past year. Free-text responses ranged from 1 to 40 and were categorized into “1”, “2” and “3+”.

##### Age at first heterosexual intercourse

Respondents were asked for their age at first sexual intercourse with an opposite-sex partner (“vaginal intercourse”; “oral sex”; “anal sex”; “other genital contact, e.g. manual contact”). Free-text responses ranged from 8 to 27 years (one person stated to have had other genital contact at the age of 8 years). Answers were categorized into “<16 years” and “16+ years” broadly corresponding to early and late sexual debut.

##### Non-use of condoms at first heterosexual intercourse

Analogous to the question about non-use of condoms at last sex (see above), a question asked about contraception use at first sexual intercourse with someone of the opposite-sex. Answers were categorized into “use of condoms” and “non-use of condoms“ (no contraception or other type of contraception).

#### Relational and factors relating to the circumstances of last sex

##### Type of relationship

Participants were asked how they perceived the relational context of their last sexual encounter (“What kind of relationship did you have with this person?”) and were offered three possible response options: “It was a one-night stand/non-recurring sex” [es war ein one-night-stand/einmaliger Sex]; “It was a longer relationship” [es war eine längere Affäre]; “It was the beginning of a loving relationship” [es war eine beginnende Liebesbeziehung]).

##### Initial meeting venue

Based on an item used in former studies,^17^ participants were asked where they had met their last sex partner. Answer categories were dichotomized into “offline” (e.g. “going out in the evening”) and “online” (e.g. “via an online dating agency”).

##### Alcohol and/or drug use at last sex

Participants were asked “The last time you had sex, were you under the influence of alcohol or drugs?” Multiple answer options were categorized into “no” (“no, neither alcohol nor drugs”), “solely alcohol” (“yes, alcohol”) and, due to low case numbers (n_unweighted_ = 31), a pooled category for “solely drugs or drugs combined with alcohol” (drug use options were: “cannabis”*;* “amyl nitrite (poppers)”*;* “a different substance”).

##### Talking about condom use before sex

With regards to their last sexual encounter, participants were asked “Did you discuss the use of condoms before having sex?” (“no”, “yes”) based on items previously used in German studies.^18^ Participants who reported not remembering (*n* = 4) were excluded from analysis.

### Statistics

The data were weighted on the basis of gender, age, education, nationality and region in accordance with the 2018 German census ahead of analyses. A detailed description of the methodology is provided by Matthiesen et al.^19^ and with further regard to the operationalization of gender by Muschalik et al.^20^

The statistical analysis was weighted using the “complex sampling” module in IBM SPSS Statistics, version 27. Descriptive frequencies are reported in conjunction with the 95% confidence interval (95%-CI). Variables hypothesised to be associated with the non-use of condoms were tested using binary logistic regression analysis. Crude odds ratios and odds ratios adjusted for gender and continuous age were reported including 95% CIs and, referring to AORs, overall *p*-values for associations.

## Results

### Sample description

Of 1,176 (unweighted) participants who reported no current relationship, 970 (82.5%) had been single for at least 1 year prior to interview. Of the subgroup who stated having had one or more sexual encounters during this time (*n* = 383), those reporting exclusively opposite-sex partners (*n* = 359, 94%) and partners of both sexes (*n* = 16, 4%) were selected. Excluding participants who did not respond to the question on contraceptive use at last sex, the analyses presented here are based on 343 participants (weighted 309), of whom 197 were men (weighted 198) and 146 women (weighted 111). Similar proportions were aged 18–35 years as they were 36–75 years, and education was evenly distributed. One-third had a migration background (Table 1).

**Table 1.** Sample characteristics.

	% (95% CI)	Denominator ǂ
Total		343, 309
Gender		
Male	64.2 (58.2–69.7)	197, 198
Female	35.8 (30.3–41.8)	146, 111
Age (years)		
18–35	46.9 (41.6–52.3)	190, 145
36–75	53.1 (47.7–58.4)	153, 164
Education		
Low	28.5 (22.5–35.3)	58, 88
Middle	32.5 (27.3–38.2)	112, 101
High	39.0 (33.2–45.1)	173, 121
Migration background		
No	71.7 (65.6–77.0)	260, 221
Yes	28.3 (23.0–34.4)	82, 87

*ǂ*Unweighted, weighted; CI: confidence interval.

### Sociodemographic characteristics

Overall, 41.6% of the participants reported not using condoms at last sex. Non-use of condoms was more commonly reported by older singles (48.5% in those aged above 35; 33.7% in those aged 18–35), but was not associated with gender or education level. In singles with a migration background not having used condoms was reported slightly less commonly (33.2 vs 45.1%), but this did not reach statistical significance at *p* < 0.05 (Table 2).

**Table 2.** Non-use of condoms at the last sex by participant characteristics.

	Non-use of condoms			
	% (95% CI)	Odds ratio (95% CI)*	*p*-value	Denominator ǂ
Total	41.6 (35.2–48.3)			343, 309
Gender				
Male	39.5 (31.8–47.8)	0.79 (0.48–1.23)	0.34	197, 198
Female	45.3 (35.8–55.2)	1.00		146, 111
Age (years)				
18–35	33.7 (26.4–41.9)	0.54 (0.32–0.91)	<0.05	190, 145
36–75	48.5 (38.7–58.5)	1.00		153, 164
Education				
Low	35.6 (24.7–48.2)	0.70 (0.40–1.21)	0.41	58, 88
Middle	43.7 (32.1–56.0)	0.92 (0.54–1.80)		112, 101
High	44.2 (36.6–52.0)	1.00		173, 121
Migration background				
No	45.1 (37.6–52.9)	1.62 (0.95–2.87)	0.07	260, 221
Yes	33.2 (23.4–44.8)	1.00		82, 87

*ǂ*Unweighted, weighted; CI: confidence interval.

**Odds ratios are based on logistic regressions.

### Sexual behaviour and sexual history

The type of sexual practice(s) was not significantly associated with reporting non-use of condoms at last sex. Participants reporting two sex partners during the past year were less likely to report sex without condom than were participants with either only one or at least three sex partners (AOR 0.54, [95% CI: 0.28–1.04]). 98% of participants reported their age at heterosexual debut to be at least 1 year younger than their age at the time the interview, giving certainty that for the majority, heterosexual debut and last sex were different sexual encounters. While age at heterosexual debut was not significantly related, non-use of condoms at that time was associated with an increased likelihood of non-use of condoms at last sex (AOR: 1.11 [95% CI: 0.64–1.93], Table 3).

**Table 3.** Non-use of condoms at the last sex in singles by sexual behaviour and sexual history factors.

	Non-use of condoms at the last sex				
Type of sexual practice during latest sex	% (95% CI)	Crude odds ratios (95% CI)	Adjusted odds ratios (95% CI)*	*p*-value	Denominator ǂ
Vaginal intercourse only	39.9 (30.3–50.4)	1.00	1.00	0.43	129, 130
Vaginal intercourse and oral sex	38.1 (28.2–49.0)	0.86 (0.45–1.65)	0.93 (0.48–1.80)		104, 87
Vaginal intercourse, oral sex and other genital contacts	53.0 (40.1–65.6)	1.58 (0.73–3.44)	1.56 (0.67–3.64)		48, 38
Other practices/combination of practices	42.0 (28.0–57.5)	1.77 (0.71–4.41)	1.72 (0.66–4.44)		58, 50
Number of sex partners during the past year	% (95% CI)				
1	44.0 (34.8–53.6)	1.00	1.00	<0.05	159, 150
2	29.6 (19.1–42.7)	0.51 (0.27–0.98)	0.54 (0.28–1.04)		79, 72
3+	47.4 (37.1–58.0)	1.28 (0.68–2.41)	1.53 (0.79–2.97)		105, 87
Age at first vaginal intercourse	% (95% CI)				
<16 years	39.8 (31.3–48.9)	1.13 (0.65–2.0)	1.11 (0.64–1.93)	0.71	128, 111
16+ years	42.6 (35.0–50.6)	1.00	1.00		215, 199
Non-use of condoms at sexual debut	% (95% CI)				
Condoms used	24.4 (18.4–31.2)	1.00	1.00	<0.001	199, 167
Non-use of condoms	62.4 (52.7–71.2)	6.12 (3.6–10.40)	4.97 (2.94–8.41)		139, 138

*ǂ*Unweighted, weighted; CI: confidence interval.

*Adjusted odds ratios are based on logistic regression adjusted for gender and age, reference category: Condoms used.

### Relational and factors relating to the circumstances of last sex

In comparison to last sex being perceived as a one-night stand/non-recurring sex, not using condoms was more likely when the sexual encounter was perceived as a longer relationship (42.7 vs 28.7%; AOR:2.43 [95% CI: 1.11–5.27]) or the beginning of a loving relationship (50.8%; AOR 3.59 [95% CI: 1.49–8.67]). Whether the sexual relation had stemmed from offline or online encounters did not seem to play a significant role in non-use of condoms, nor did sex-related substance use. Absent communication about condom use before the sex was associated with an increased likelihood for non-use of condoms (67.5%; AOR 6.50 [95% CI: 3.24–13.04]; Table 4).

**Table 4.** Non-use of condoms at last sex in singles by relational and factors relating to the circumstances of last sex.

	Non-use of condoms at last sex				
	% (95% CI)	Crude odds ratios (95% CI)	Adjusted odds ratios (95% CI)*	*p*-value	Denominator ǂ
Context of the sexual encounter					
One-night stand/non-recurring sex	28.7 (20.6–38.5)	1.00	1.00	<0.01	108, 94
Longer relationship	42.7 (33.6–52.4)	2.40 (1.23–4.66)	2.43 (1.11–5.27)		113, 94
Beginning of a love relationship	50.8 (36.2–65.3)	3.74 (1.48–9.46)	3.59 (1.49–8.66)		64, 66
Initial meeting venue					
Offline	41.4 (34.2–49.0)	1.00	1.00	0.742	249, 222
Online	41.6 (28.4–56.2)	1.18 (0.56–2.49)	0.89 (0.43–1.83)		69, 61
Alcohol and/or drug use at last sex					
No	46.2 (37.5–55.1)	1.00	1.00	0.719	174, 159
Yes, alcohol	33.8 (25.2–43.6)	0.83 (0.40–1.73)	0.98 (0.45–2.15)		137, 121
Yes, drugs/drugs + alcohol	45.7 (30.1–62.2)	0.93 (0.30–2.26)	1.43 (0.58–3.51)		31, 28
Talked about condom use before last sex					
No	67.5 (56.3–77.00)	6.59 (3.21–13.50)	6.50 (3.24–13.04)	<0.001	116, 106
Yes	27.7 (21.9–34.5)	1.00	1.00		223, 200

*ǂ*Unweighted, weighted; CI: confidence interval.

*Adjusted odds ratios are based on logistic regression adjusted for gender and age; reference category: Condoms used.

## Discussion

The present study analysed data from a survey designed to be broadly representative of the German population to investigate factors associated with reporting non-use of condoms at last sex among heterosexually-active single people in Germany. The prevalence of not using condoms in this population was estimated as over 40%. Although previous studies have used different measures of non-use of condoms, including over longer timeframes and for younger populations, our finding is consistent in that non-use of condoms remains common in Germany.^10,18^

Non-use of condoms was more prevalent in people in mid- and older life than in younger ages, a finding observed in numerous international studies on general condom-use, too.^8,9,11,12,21^ The relatively low importance of needing to prevent unintended pregnancies in middle-aged and older adults,^10^ as well as less awareness and/or knowledge of STI risks relative to the younger generation, as indicated by another analysis within the GeSiD-study,^22^ and reflecting how interventions including STI screening predominantly target younger people,^3^ are possible explanations for greater sexual risk-taking in advanced ages at least according to this measure. In large studies from other countries, gender,^23^ education^11,24^ as well as ethnicity in women^8,11^ were shown to be associated with condom-use. In contrast, in the present sample of specifically heterosexually-active singles’, condom-use at last sex was not found to be associated with gender, education and migration background. Whether these findings reflect the limited statistical power of the present analysis or turn out to be population-specific, should be investigated in future studies.

Non-use of condoms was more likely in the course of longer relationships (“längere Affäre”) or early loving relationships (“beginnende Liebesbeziehung”) than in one-night-stands (“one-night-stand/einmaliger Sex”), indicating that the duration of an acquaintance, as well as perceived closeness, familiarity and commitment may play a role in deciding whether to use condoms, in line with previous research.^12,23,24^ Not having talked about using condoms before sex was associated with the non-use of condoms. These two aspects may be related with each other considering findings from Peasant et al.^25^ who showed that talking about condom use prior to sex seemed to be more difficult with growing familiarity in relationships.

Unlike findings from other studies,^5,8,9^ age at heterosexual debut did not seem to play a role, but not having used condoms at that time was associated with an increased likelihood for non-use of condoms at last sex. This appeared consistent with findings from other countries^5,9^ and supports the idea that routinely using condoms from the start of one’s sexual career may promote the adoption of safer sex practices at least in terms of condom use throughout adulthood.^26,27^

Findings from other countries which identified a large number of sexual partners^4–6,28^ as a risk factor for the non-use of condoms were replicated in our study. Interestingly, in our study the association between the number of sex partners and the likelihood for non-use of condoms appeared to be nonlinear: Having had two sex partners during the past year was associated with a lower likelihood for the non-use of condoms than having had only one or either three or more sex partners.

Whether participants had engaged in only one or several sexual practices did not affect the likelihood for non-use of condoms in the current study, which stands in contrast to findings by Hill et al.,^29^ who found that switching practices during a sexual encounter, for example between vaginal intercourse and oral stimulation, was critical for having condomless sex. However, it cannot be gathered from the questions asked about condoms in this study, whether a condom had been used continuously or removed during the sexual act. Further studies should look to ask more detailed questions about condom use in order to help identify and understand potentially risky situations.

Meeting the partner online or offline was not found to be associated with non-use of condoms, indicating that the likelihood for having condomless sex in Germany is not higher in online initiated contacts, in line with findings from Great Britain.^28^ A closer look at online venue preferences and personal motivations (seeking casual sex or romantic relationships) might help gain further insights into different patterns of sexual risk taking.^28,30^ While sex-related alcohol consumption played a role in nearly 40% of the heterosexual singles’ sexual encounters in the present study, there was no association with the non-use of condoms, unlike other studies suggest.^7,29^

### Limitations

Among those participants identified as being single for at least 1 year prior to interview, almost 40% reported sexual activity with someone of the opposite-sex during this timeframe. On the one hand, this means that the majority of singles live a “healthy” lifestyle in terms of STI risk simply by having no sex at all. On the other hand, the statistical power for the study’s examination of those heterosexually-active singles is limited. While we tried to increase the statistical power available, e.g. using only binary categories in our analyses, it is likely that we had limited ability to detect as statistically significant differences that may be of clinical or public health significance. Further analysis on larger samples is needed, enabling a greater granularity, for example by age, type of online meeting venue, and type of drug used. They should also to take account of specific health conditions, in particular having a current or recent STI-diagnosis which has been shown to be associated with condom use^11^ but which could not be considered in the present study due to the small number reporting a STI diagnosis (7.4%, *n* = 24). Furthermore, with survey data like these it is not possible to determine chronology, i.e. whether the diagnosis was before or after last sex.

Findings are limited to the reports about condom-use during a single occasion, namely the last sex, allowing us to identify specific correlates and circumstances related to this particular event. It may be helpful for future studies to consider consistency of condom-use over a longer timeframe to shed further light on the mechanisms underlying risky behaviour. For example, with longer acquaintances, negotiations about condom use may only take place prior to the initial sexual encounter, such that they are not repeated in subsequent sexual encounters with the sex partner. Future studies need to highlight those factors that support ongoing discussion about condoms in relationships with growing familiarity.

## Conclusions

Health promotion continues to be needed to promote the importance of condom-use in Germany, especially in the context of sex with a new partner from sexual debut and throughout the life course, regardless of relationship type, age, and relatedly, concerns around pregnancy risk. Improving skills for fear-free and natural communication around condom use and sexual interactions more broadly should be part of interventions promoted through public awareness campaigns and in individual counselling.
